# Automated versus non-automated weaning for reducing the duration of mechanical ventilation for critically ill adults and children: a cochrane systematic review and meta-analysis

**DOI:** 10.1186/s13054-015-0755-6

**Published:** 2015-02-24

**Authors:** Louise Rose, Marcus J Schultz, Chris R Cardwell, Philippe Jouvet, Danny F McAuley, Bronagh Blackwood

**Affiliations:** Department of Critical Care Medicine, Sunnybrook Health Sciences Centre, 2075 Bayview Ave, Toronto, ON M4N 3M5 Canada; Lawrence S. Bloomberg Faculty of Nursing, University of Toronto, 155 College St, Toronto, Ontario M5T IP8 Canada; Provincial Centre of Weaning Excellence, Toronto East General Hospital, Toronto, Canada; Li Ka Shing Institute, St Michael’s Hospital, 30 Bond St, Toronto, ON M5B 1W8 Canada; West Park Healthcare Centre, University of Toronto, 155 College St, Toronto, Ontario M5T IP8 Canada; Laboratory of Experimental Intensive Care and Anesthesiology, Academic Medical Center, University of Amsterdam, Meibergdreef 9, 1105 AZ Amsterdam, Netherlands; Centre for Public Health, Queen’s University Belfast, University Rd, Belfast, BT7 1NN UK; Department of Pediatrics, Sainte-Justine Hospital, University of Montreal, 3175 Chemin de la Côte-Sainte-Catherine, Montreal, QC H3T 1C5 Canada; Regional Intensive Care Unit, Royal Victoria Hospital, Centre for Infection and Immunity, Queen’s University of Belfast, University Rd, Belfast, BT7 1NN UK; Centre for Infection and Immunity, School of Medicine, Dentistry & Biomedical Sciences, Queen’s University Belfast, University Rd, Belfast, BT7 1NN UK

## Abstract

**Introduction:**

Automated weaning systems may improve adaptation of mechanical support for a patient’s ventilatory needs and facilitate systematic and early recognition of their ability to breathe spontaneously and the potential for discontinuation of ventilation. Our objective was to compare mechanical ventilator weaning duration for critically ill adults and children when managed with automated systems versus non-automated strategies. Secondary objectives were to determine differences in duration of ventilation, intensive care unit (ICU) and hospital length of stay (LOS), mortality, and adverse events.

**Methods:**

Electronic databases were searched to 30 September 2013 without language restrictions. We also searched conference proceedings; trial registration websites; and article reference lists. Two authors independently extracted data and assessed risk of bias. We combined data using random-effects modelling.

**Results:**

We identified 21 eligible trials totalling 1,676 participants. Pooled data from 16 trials indicated that automated systems reduced the geometric mean weaning duration by 30% (95% confidence interval (CI) 13% to 45%), with substantial heterogeneity (I^2^ = 87%, *P* <0.00001). Reduced weaning duration was found with mixed or medical ICU populations (42%, 95% CI 10% to 63%) and Smartcare/PS™ (28%, 95% CI 7% to 49%) but not with surgical populations or using other systems. Automated systems reduced ventilation duration with no heterogeneity (10%, 95% CI 3% to 16%) and ICU LOS (8%, 95% CI 0% to 15%). There was no strong evidence of effect on mortality, hospital LOS, reintubation, self-extubation and non-invasive ventilation following extubation. Automated systems reduced prolonged mechanical ventilation and tracheostomy. Overall quality of evidence was high.

**Conclusions:**

Automated systems may reduce weaning and ventilation duration and ICU stay. Due to substantial trial heterogeneity an adequately powered, high quality, multi-centre randomized controlled trial is needed.

**Electronic supplementary material:**

The online version of this article (doi:10.1186/s13054-015-0755-6) contains supplementary material, which is available to authorized users.

## Introduction

Serious physiological and psychological sequelae are associated with protracted invasive mechanical ventilation, necessitating efficient processes to safely reduce and remove ventilator support, termed weaning [1,2]. Tools such as weaning protocols and automated systems may facilitate systematic and early recognition of spontaneous breathing ability and the potential for ventilation discontinuation. These tools may reduce practice variation and improve efficiency by emphasizing timely and objective decision making [3]. A 2010 Cochrane review evaluating the effectiveness of protocolized versus non-protocolized weaning [4] found evidence of reduced duration of mechanical ventilation, weaning and intensive care unit (ICU) stay using standardized weaning protocols but also significant heterogeneity among studies.

Weaning from mechanical ventilation traditionally occurs via clinician-directed adjustments to the level of assistance provided by the ventilator culminating in a spontaneous breathing trial. Through continuous monitoring and real-time interventions, automated systems theoretically provide improved adaptation of ventilatory support to patients’ needs when compared to clinician-directed weaning [5]. Several such systems are now commercially available and include Smartcare/PS™ (DrägerMedical, Lübeck, Germany), Adaptive Support Ventilation (ASV) (HamiltonMedical, Bonaduz, Switzerland), Automode (Siemens, Solna, Sweden), Proportional Assist Ventilation (PAV+ The University of Manitoba, Canada used under license by Covidien, Minneapolis, US), Mandatory Minute Ventilation (MMV) (Dräger Medical), Proportional Pressure Support (PPS) (DrägerMedical), Neurally Adjusted Ventilatory Assist (NAVA) (Maquet, Solna, Sweden) and Intellivent-ASV®(Hamilton Medical, Rhäzüns, Switzerland). (see Additional file 1). Automation of weaning potentially reduces avoidable delays as it is less reliant on clinician recognition of changes in the patient’s weaning status which can be influenced by clinician availability, expertise and work load.

There is a pressing imperative to identify efficiencies in weaning resulting in reduced ventilation duration to prevent ventilator associated morbidity and mortality, and also to offer solutions to existing constraints in critical care services. The number of patients receiving mechanical ventilation continues to increase due to improved patient survival and population aging [6]. Costs of care provision to these patients are substantial [7]. This increased demand is occurring alongside a reduced supply of healthcare professionals qualified and skilled in mechanical ventilation management and its weaning [8,9]. If efficacious in terms of clinical outcomes, automated weaning systems could enable safe and efficient management of weaning despite predicted staffing shortages. Therefore, our primary objective was to compare the total weaning duration, defined as study randomization to successful extubation for critically ill ventilated adults and children managed with an automated weaning system versus non-automated strategies. Secondary objectives were to determine differences in ventilation duration, ICU and hospital lengths of stay (LOS), mortality and adverse events related to early or delayed extubation. More details regarding this review can be found in the Cochrane Database of Systematic Reviews [10].

## Methods

Trials were eligible if they were randomized, compared automated systems designed to reduce ventilator support based on continuous monitoring of patient tolerance and interpretation of real-time physiological changes to non-automated weaning strategies including standard or usual care and protocolized weaning, and were conducted in critically ill adults and children over four weeks of age. We included adults and children as the same tenets of weaning apply [11]. Research Ethics Board approval and participant consent were not required as this is a systematic review including published and non-published data from existing trials.

Two authors independently searched MEDLINE, EMBASE, Cochrane Central Register of Controlled Trials, CINAHL, LILACS, Web of Science, the Database of Abstracts of Reviews of Effects and the Health Technology Assessment Database up to 30 September 2013. We combined search terms for automated systems with the Cochrane highly sensitive search strategy for randomized controlled trials; language restrictions did not apply. We searched conference proceedings, trial registration websites, reference lists of retrieved studies and review papers and contacted trial authors and content experts.

### Data extraction

Using a standardized form, two authors independently extracted: study design and setting, participant characteristics, inclusion/exclusion criteria, weaning methods (intervention and control arms), sedation strategies and study outcomes. We recorded randomization methods, allocation concealment, blinding, frequency and handling of missing data, adherence to intention-to-treat analysis, and selective outcome reporting. We contacted corresponding authors to seek further clarification on issues of reporting or to obtain additional outcome data. Data extractors were not blinded to study citations.

### Assessment of methodological quality

Two authors independently assessed methodological quality and risk of bias using the domain-based evaluation recommended by The Cochrane Collaboration [12]. Domains judged as high, low or unclear risk of bias included: random sequence generation, allocation concealment, blinding, incomplete outcome data, selective reporting and other biases. We used assessment of risk of bias to perform sensitivity analyses based on methodological quality.

### Statistical analyses

We calculated the difference in means, 95% confidence intervals (CIs) and the standard error of that difference for continuous outcomes. For dichotomous data, we described treatment effects using risk ratios (RR) and 95% CIs. We used random-effects models to calculate pooled estimates as heterogeneity was anticipated [13]. As we detected considerable skew in continuous outcomes, we log transformed data for the primary analysis. For six trials [14-19], we log transformed raw data obtained from corresponding authors. For nine trials [20-28], we used the mean and standard deviation (SD) on the unlogged scale to calculate a mean and SD on the log transformed scale using methods described by Higgins [12]. For six trials [29-34], we used the median and interquartile range (IQR) of the unlogged data to approximate the mean if not available using methods described by Hozo and colleagues [35] and calculated an approximate SD on the log scale from the IQR on the log scale [12]. We exponentiated the difference in the mean of a variable on the log scale to provide the ratio of geometric means on the unlogged scale and reported this as a percentage change and 95% CIs (reduction or increase) in geometric mean for ease of understanding [36]. All analyses were performed in Review Manager 5.2 [37].

### Subgroup and sensitivity analyses

Pre-specified subgroup analyses were performed based on ICU population grouped as either ‘medical and mixed’ or ‘surgical’; the automated system evaluated; and the non-automated weaning strategy used (protocolized versus non-protocolized weaning). Due to limited numbers of studies we collapsed studies of medical or mixed ICU population into one group. We were unable to perform *a priori* planned subgroup analyses according to weaning classification (simple, difficult, prolonged [2]) as no trials used this taxonomy; adult versus pediatric populations as only one trial recruited children; and sedation strategy as this was inadequately reported in most trials. We conducted sensitivity analyses on unlogged data for all continuous outcomes and on logged data for the primary outcome excluding trials with high risk of bias.

### Assessment of heterogeneity and reporting bias

We evaluated clinical heterogeneity by qualitative assessment of study differences in terms of study population, ICU type, clinician involvement in weaning decision making, and implementation of weaning and extubation processes. Statistical heterogeneity was informally evaluated from forest plots, and more formally using the Chi square test (*P* <0.05, significant heterogeneity) and I^2^ statistic (I^2^ > 50%, moderate to substantial heterogeneity) [12]. We constructed a funnel plot of the treatment effect for the primary outcome against trial precision (standard error). We identified sufficient studies (≥10) to formally test for asymmetry [38].

## Results

**Figure 1:**
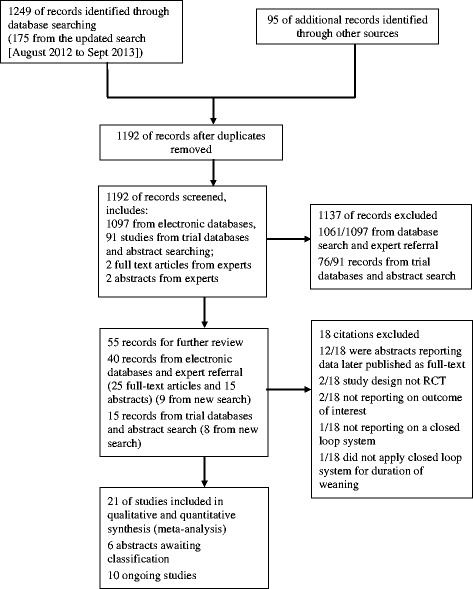
**Study flow diagram.**

**Figure 2:**
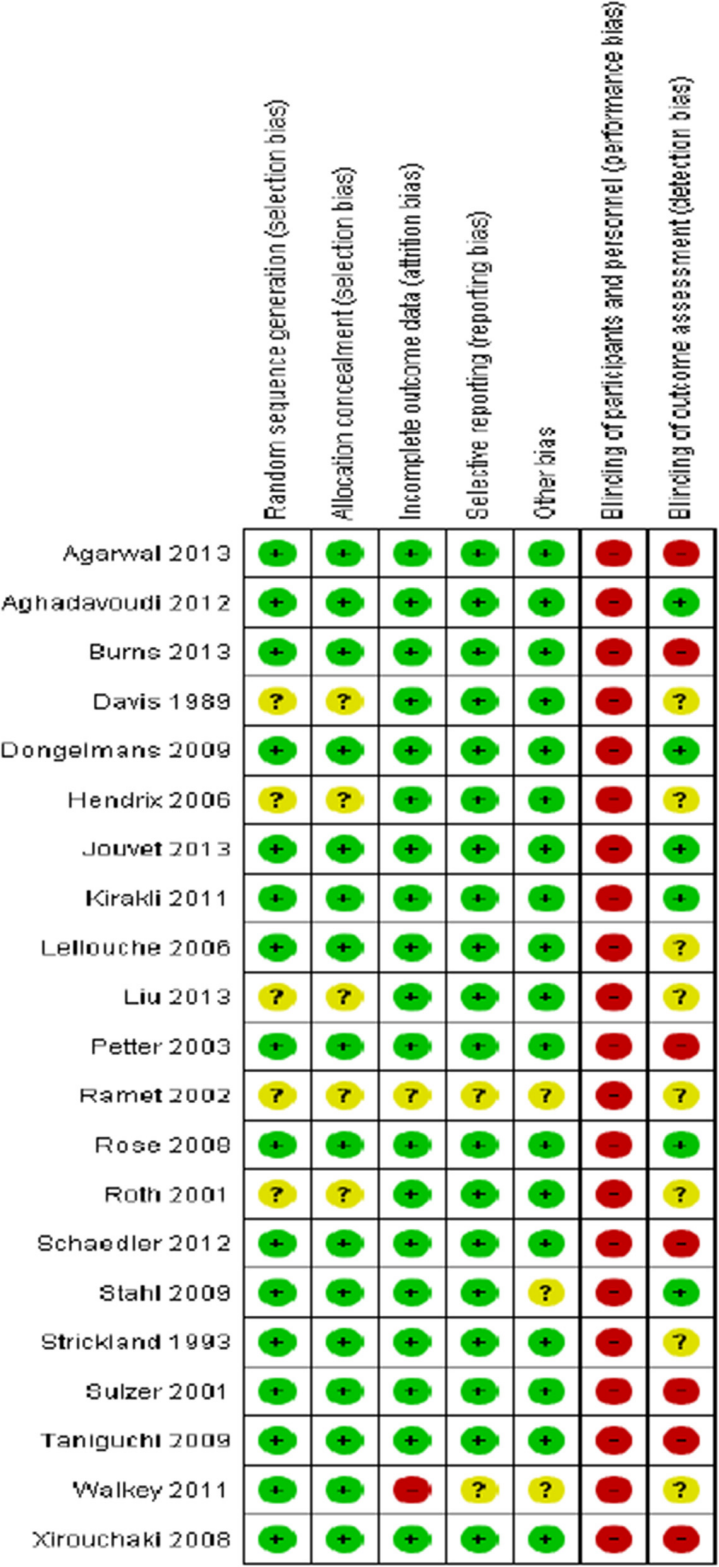
**Risk of bias summary.**

Of the retrieved 1,249 citations, 21 trials of 1,676 participants were included (Figure 1). Twelve trials recruited mixed or medical ICU populations (n = 871, 52%) [14-16,18,21,23,25,27-31] and nine surgical ICU patients (n = 805, 48%) [17,19,20,22,24,26,32-34]. Eight trials evaluated Smartcare/PS™ (DrägerMedical) (n = 803, 48%) [14,16,17,23,27,30,31,33], seven trials Adaptive Support Ventilation (ASV) (HamiltonMedical) (n = 424, 25%) [15,19,20,29,32,34], three trials Automode (Siemens) [22,24,28] (n = 78. 5%), one trial Mandatory Minute Ventilation DrägerMedical) [21], one trial Mandatory Rate Ventilation [26], one trial Proportional Assist Ventilation (PAV^+^) [18], and one trial describing a non-commercial automated system [25]. Sixteen trials [15,17-24,26,28-32,34] used protocolized weaning as the comparator; five [10,16,25,27,33] used usual care (Table 1). Most studies were of high methodological quality (Figure 2) although no studies blinded participants or clinical personnel.

**Table 1:** **Characteristics of included studies**

**Study**	**Patients**	**System**	**Comparator**	**Weaning success**
Agarwal, 2013 [29]	48 adults	ASV	Protocolized: VCV to PSV	Not defined
Aghadavoudi, 2012 [20]	81 adults	ASV	Protocolized:	Not defined
			SIMV (VC) + PS to CPAP	
Burns, 2013 [30]	92 adults	Smartcare/PS	Protocolized: PSV	Not defined although outcomes reported for first and successful extubation
Davis, 1989 [21]	40 adults	MMV	Protocolized: IMV to CPAP	Not defined
Dongelmans, 2009 [19]	128 adults	ASV	Protocolized: PCV to PSV	Tracheal extubation
Hendrix, 2006 [22]	20 adults	Automode	Protocolized: PRVC to PSV	Not defined
Jouvet, 2013 [14]	30 children	Smartcare/PS	Usual care: PSV	No further need for ventilation for 48 hours after extubation
Kirakli, 2011 [15]	97 adults	ASV	Protocolized:	No further need for ventilation for 48 hours after extubation or with a tracheostomy cannula at day 28
			AC (VC) to PSV	
Lellouche, 2006 [23]	147 adults	Smartcare/PS	Protocolized: PSV or T-piece	Time from inclusion until successful extubation (followed by 72 hours without ventilator support)
Liu, 2013 [31]	39 adults	Smartcare/PS	Protocolized: daily 30 min SBT of CPAP or PSV	48 hours without reintubation after extubation
Petter, 2003 [32]	34 adults	ASV	Protocolized: SIMV to PSV	Not defined
Ramet, 2002 [28]	18 children	Automode	Protocolized: PRVC to VS	Not defined
Rose, 2008 [16]	102 adults	Smartcare/PS	Usual care: PSV	No further need for ventilation for 48 hours after extubation
Roth, 2001 [24]	40 adults	Automode	Protocolized: SIMV to PSV	Not defined
Schädler, 2012 [17]	300 adults	Smartcare/PS	Protocolized: PSV	Not defined though outcomes reported for first and successful extubation
Stahl, 2009 [33]	60 adults	Smartcare/PS	Usual care: PSV	Measured reintubation frequency within 48 hours of extubation
Strickland, 1993 [25]	17 adults	Non-commercial system	Usual care: SIMV to PSV/CPAP	Not defined
Sulzer, 2001 [34]	36 adults	ASV	Protocolized: SIMV to PSV	Not defined
Taniguchi, 2009 [26]	106 adults	MRV	Protocolized: PCV to PSV	Not defined
Walkey [27]	33 adults	Smartcare/PS	Usual care: A/C to PSV	No further need for ventilation for 48 hours after extubation
Xirouchaki, 2008 [18]	208 adults	PAV+	Protocolized: PSV	Not defined

ASV, adaptive support ventilation; CPAP, continuous positive airway pressure; IMV, intermittent mandatory ventilation; MRV, minute respiratory volume; PAV+, proportional assist ventilation; PRVC, pressure regulated volume control; PSV, pressure support ventilation; SBT, spontaneous breathing trial; SIMV, synchronized intermittent mandatory ventilation; VS, volume support.

### Weaning duration

**Figure 3:**
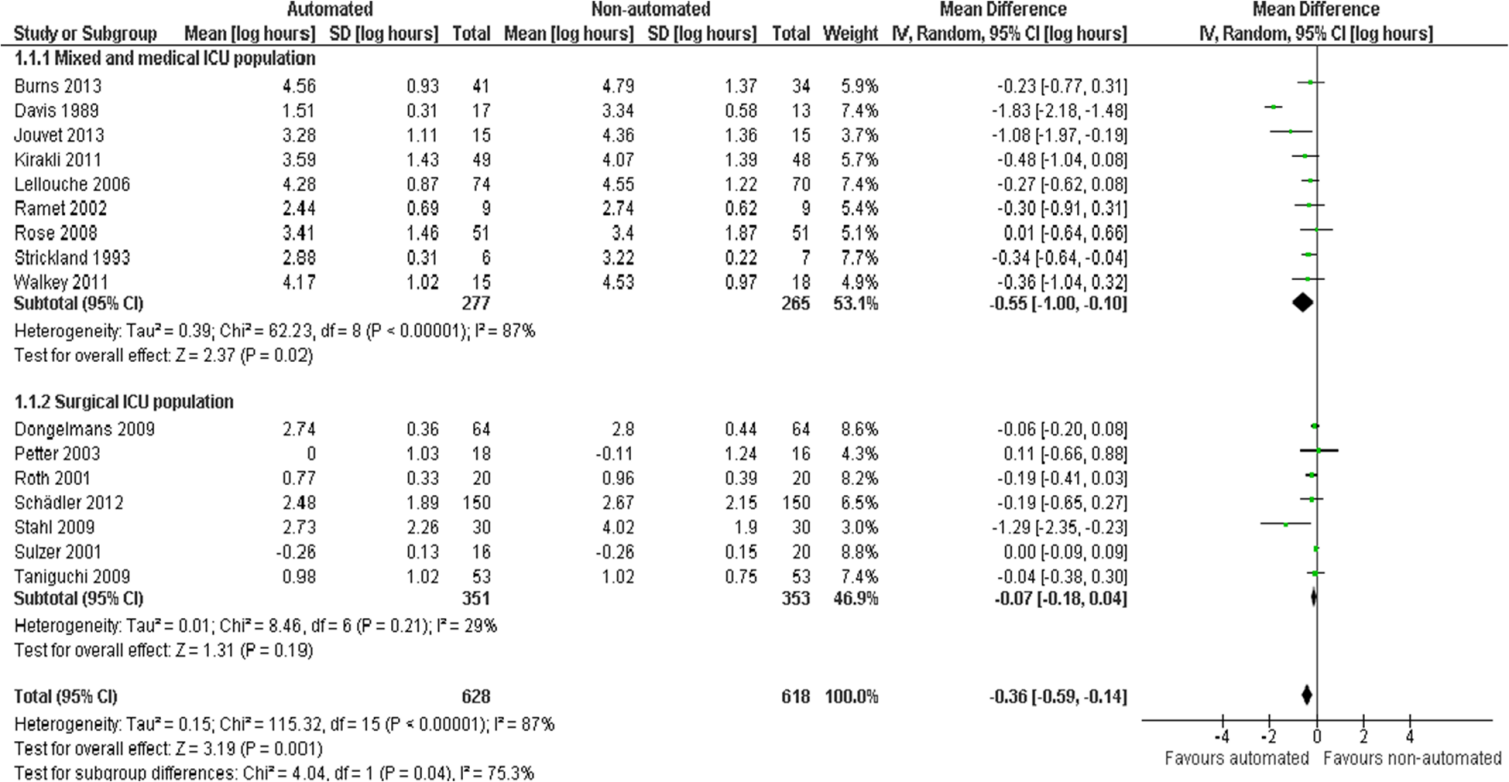
**Duration of weaning by study population.**

**Figure 4:**
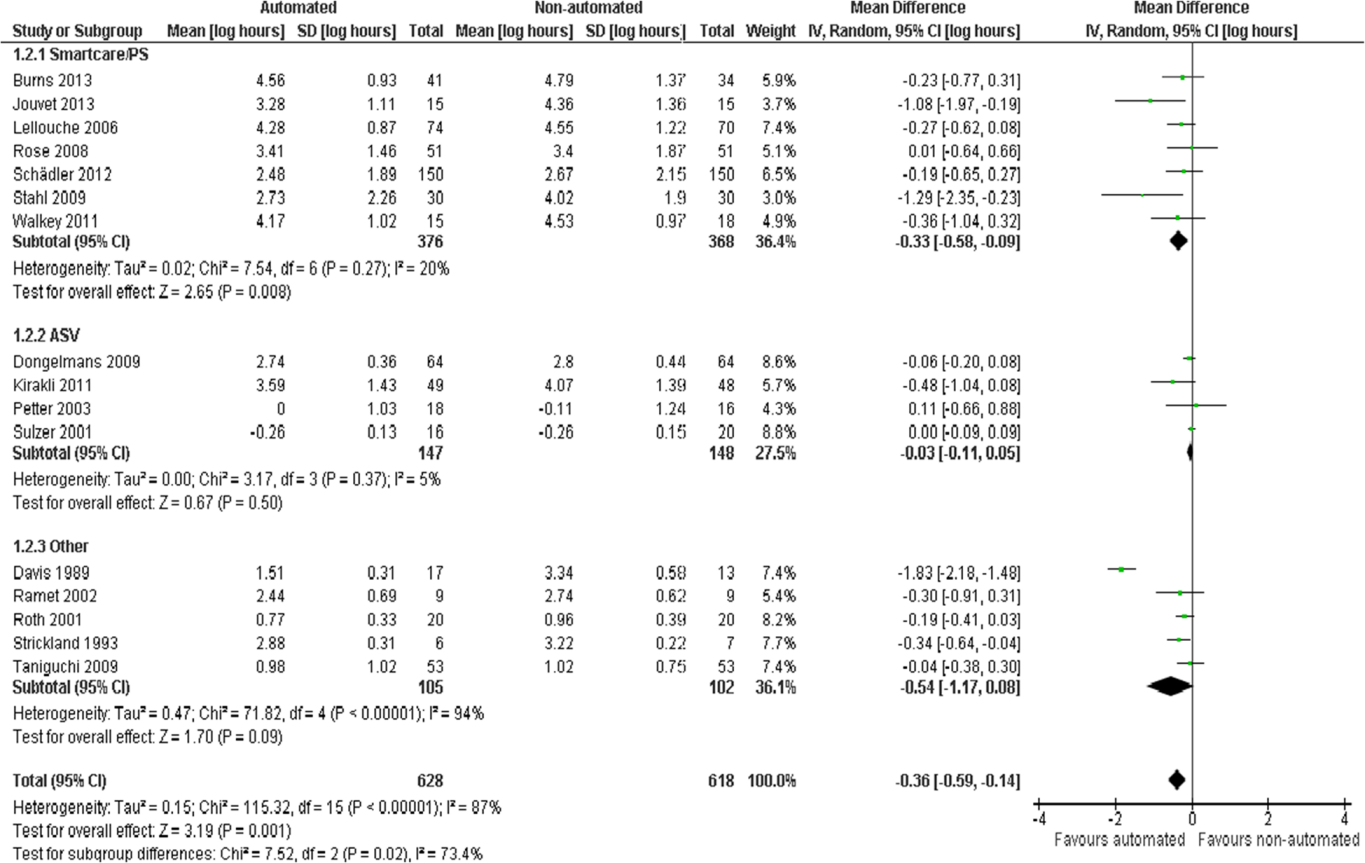
**Duration of weaning by automated system.**

Pooled data from 16 trials reporting weaning duration indicated a reduction using automated systems (mean log hours −0.36, 95% CI −0.59 to −0.14, *P* = 0.001), equivalent to a 30% (95% CI 13% to 45%) reduction in the geometric mean. Statistically significant (*P* <0.00001) and substantial (*I*^2^ = 87%) heterogeneity was noted (Figure 3). Subgroup analyses according to ICU population demonstrated reduced weaning duration in trials of mixed/medical ICU patients (mean log hours −0.55, −1.00 to −0.10, *P* = 0.02; 42% (10% to 63%) reduction in geometric mean). No evidence of effect was found in trials including only surgical ICU patients. Smartcare/PS™ reduced weaning duration (mean log hours −0.33, −0.58 to −0.09, *P* = 0.008; 28% (7% to 49%) reduction in geometric mean) whereas ASV (mean log hours −0.03, −0.11 to 0.05, *P* = 0.50; 3% (5% increase to 10% reduction) reduction in geometric mean) and other systems (mean log hours −0.54, −1.17 to 0.08, *P* = 0.09; 42% (8% increase to 69% reduction) reduction in geometric mean) did not (Figure 4). There was no subgroup difference according to the weaning method used in the control arm with broadly overlapping CIs.

### Randomization to first extubation

Pooled data from eleven trials reporting duration of study randomization to first extubation (as opposed to successful extubation used for weaning duration) demonstrated a reduction favouring automated systems (mean log hours −0.20, −0.34 to −0.05, *P* = 0.007; 18% (5% to 29%) reduction in geometric mean), although statistically significant (*P* = 0.0005) and substantial (*I*^2^ = 68%) heterogeneity was present.

### Ventilation duration

**Figure 5:**
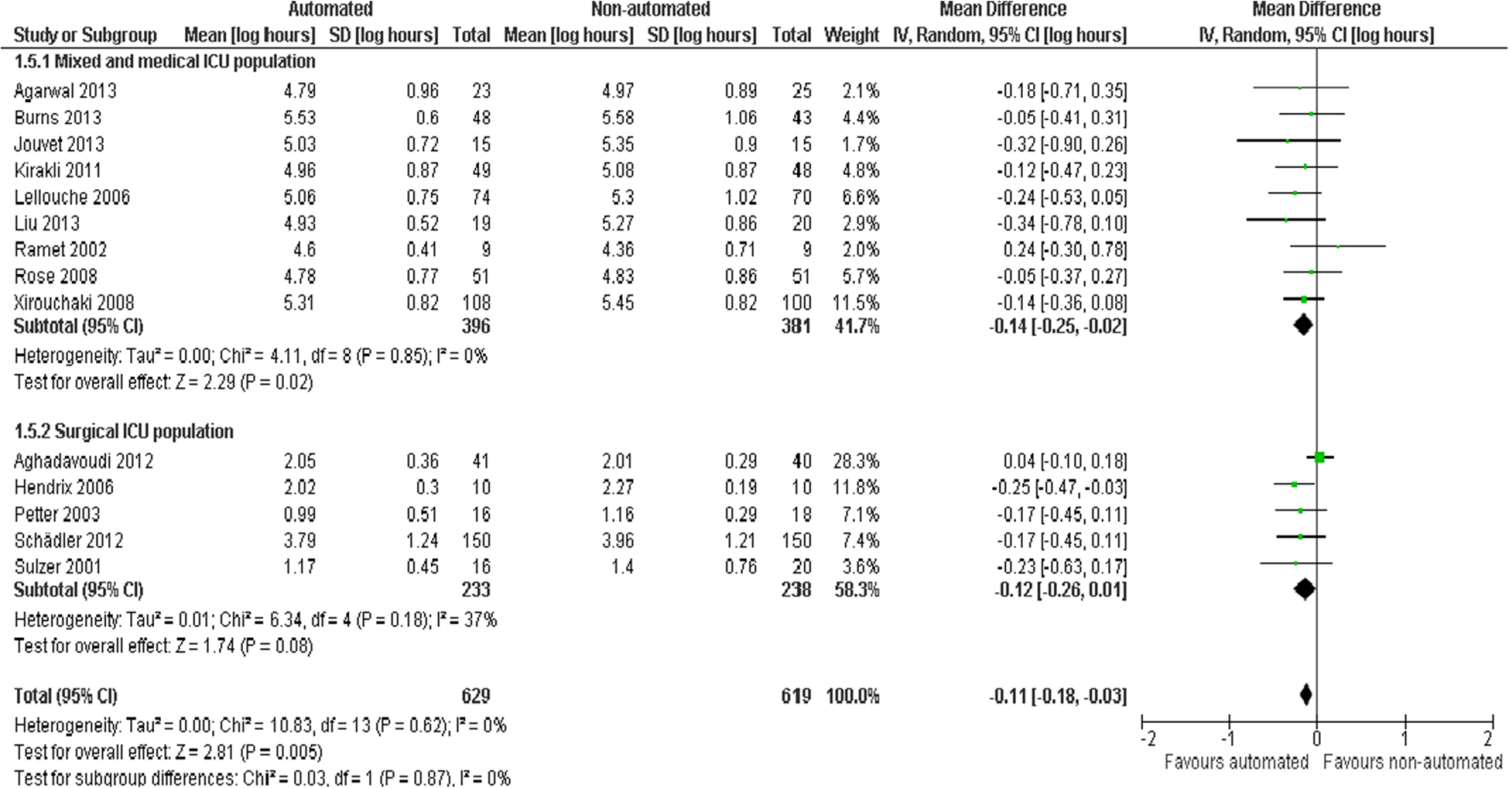
**Duration of ventilation by automated system.**

Pooled data from 14 trials reporting total ventilation duration indicated a reduction favouring automated systems (mean log hours −0.11, −0.18 to −0.03, *P* = 0.005; 10% (3% to 16%) reduction in geometric mean) with no heterogeneity (*I*^2^ = 0%, *P* = 0.62) (Figure 5). There were no subgroup differences according to ICU population, automated system, or the weaning method used in the control arm with broadly overlapping CIs for each subgroup comparison.

### Mortality

**Figure 6:**
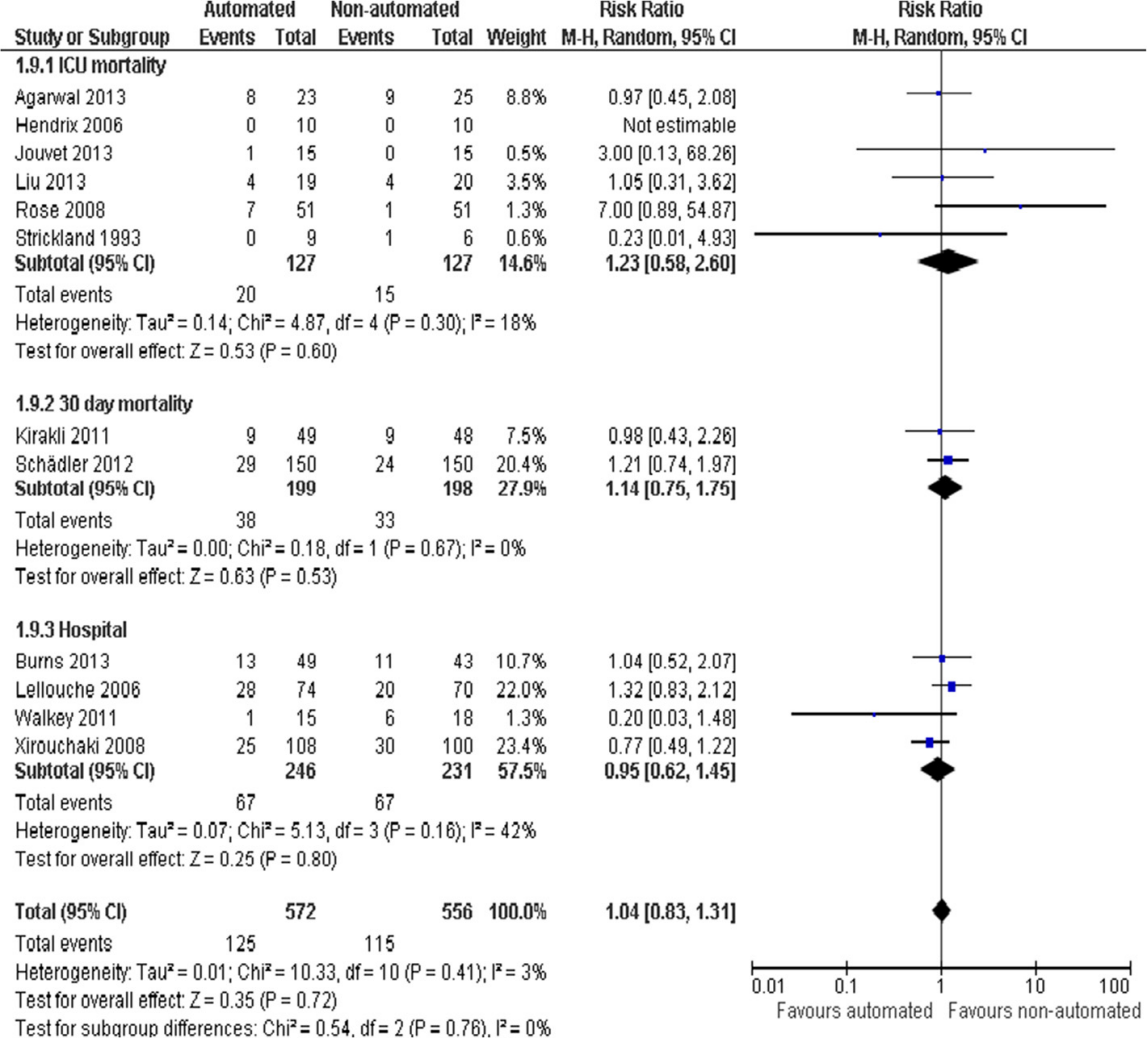
**Mortality.**

Due to relatively wide CIs (RR 1.04, 0.83 to 1.31, *P* = 0.72), pooled data from 12 trials did not provide strong evidence that automated systems had an effect on mortality when compared to non-automated weaning. Little heterogeneity was noted (*I*^2^ = 3%) (Figure 6).

### Duration of hospital and ICU stay

**Figure 7:**
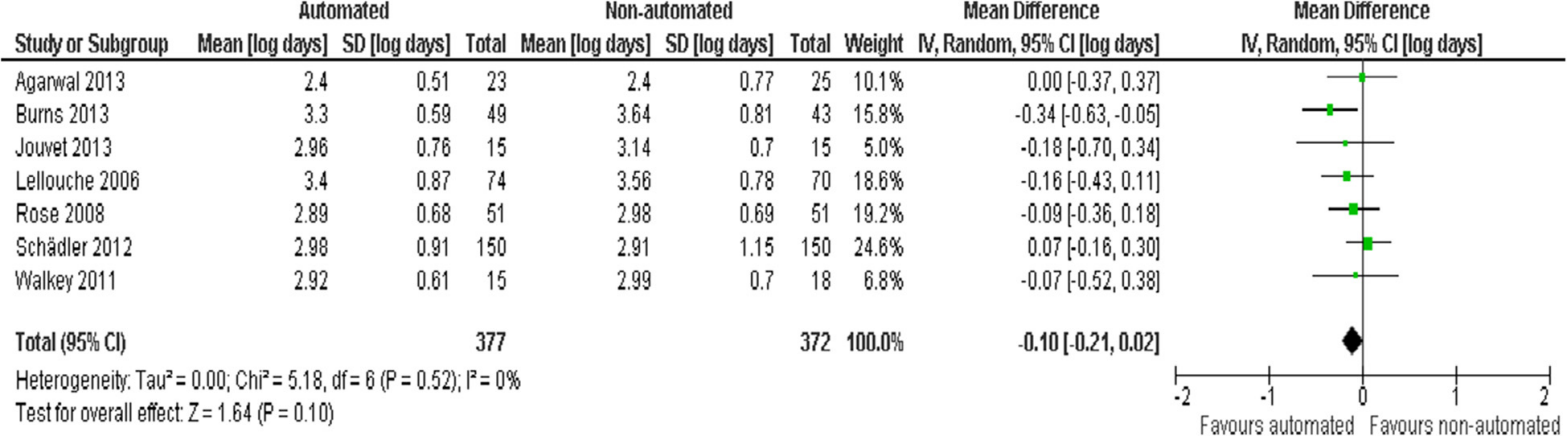
**Hospital length of stay.**

**Figure 8:**
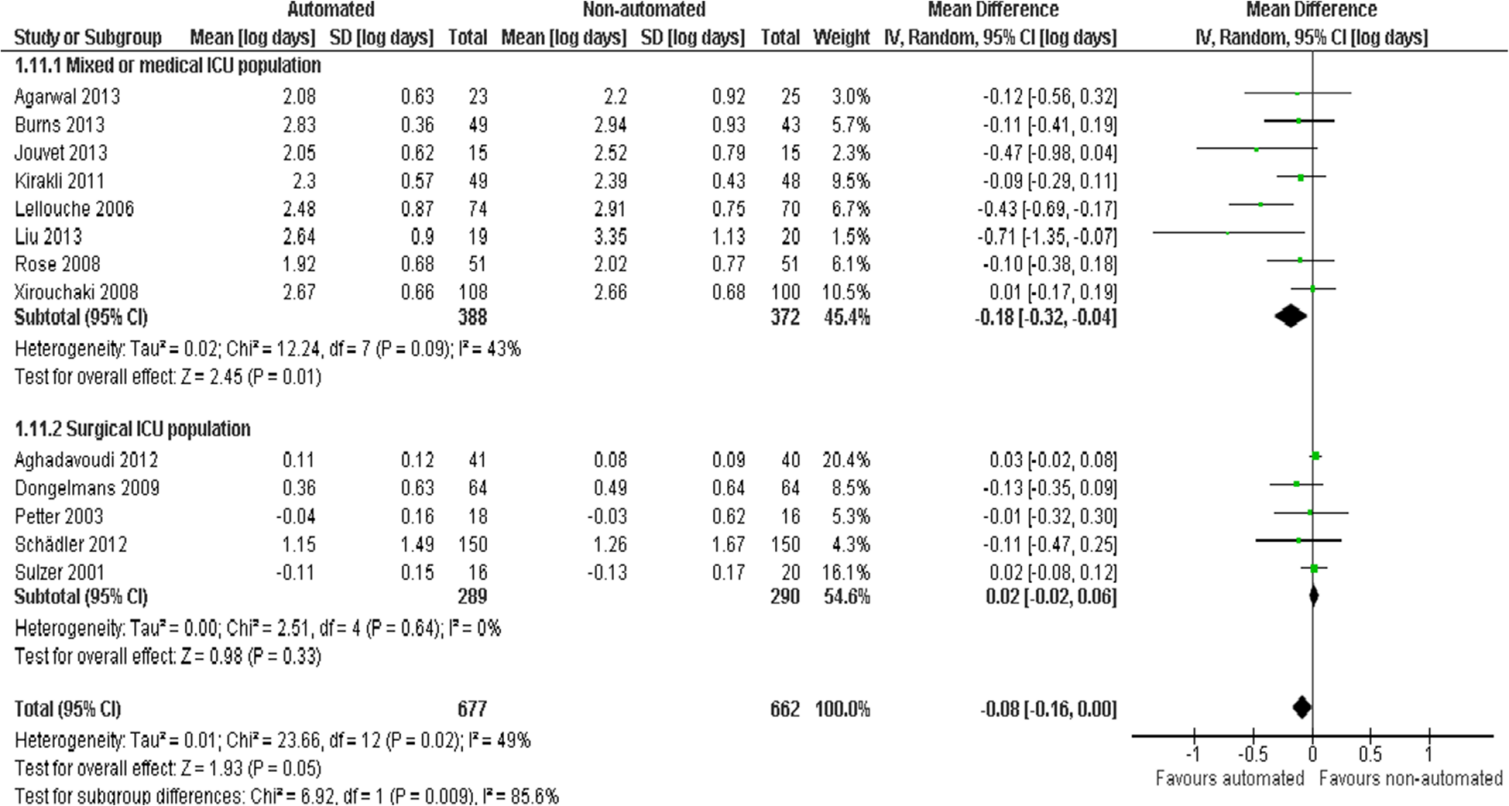
**ICU length of stay by study population.**

Pooled data from seven trials reporting hospital LOS found no evidence of effect comparing automated systems and non-automated weaning (mean log days −0.10, −0.21 to 0.02, *P* = 0.10; 6% (2% increase to 19% reduction) reduction in geometric mean) with no heterogeneity (*I*^2^ = 0%, *P* = 0.52) (Figure 7). Pooled data from 13 trials reporting ICU LOS demonstrated a reduction favouring automated systems (mean log days −0.08, −0.16 to 0.00, *P* = 0.05; 8% (0% to 15%) reduction in geometric mean) with moderate heterogeneity (*I*^2^ = 49%, *P* = 0.02). Pooled data from studies conducted in mixed/medical ICU populations demonstrated reduced ICU stay (mean log days −0.18, −0.32 to −0.04, *P* = 0.01; 15% (4% to 25%) reduction in geometric mean) but trials in surgical ICU populations did not (mean log days 0.02, − 0.02 to 0.06, *P* = 0.29; 2% (2% reduction to 6% increase) increase in geometric mean) (Figure 8). Pooled data from Smartcare/PS™ trials identified reduced ICU stay (mean log days −0.26, −0.43 to −0.09, *P* = 0.003; 23% (9% to 35%) reduction in geometric mean) whereas ASV trials did not (mean log days 0.02, −0.02 to 0.06, *P* = 0.39; 2% (2% reduction to 6% increase) increase in the geometric mean).

### Adverse events

**Figure 9:**
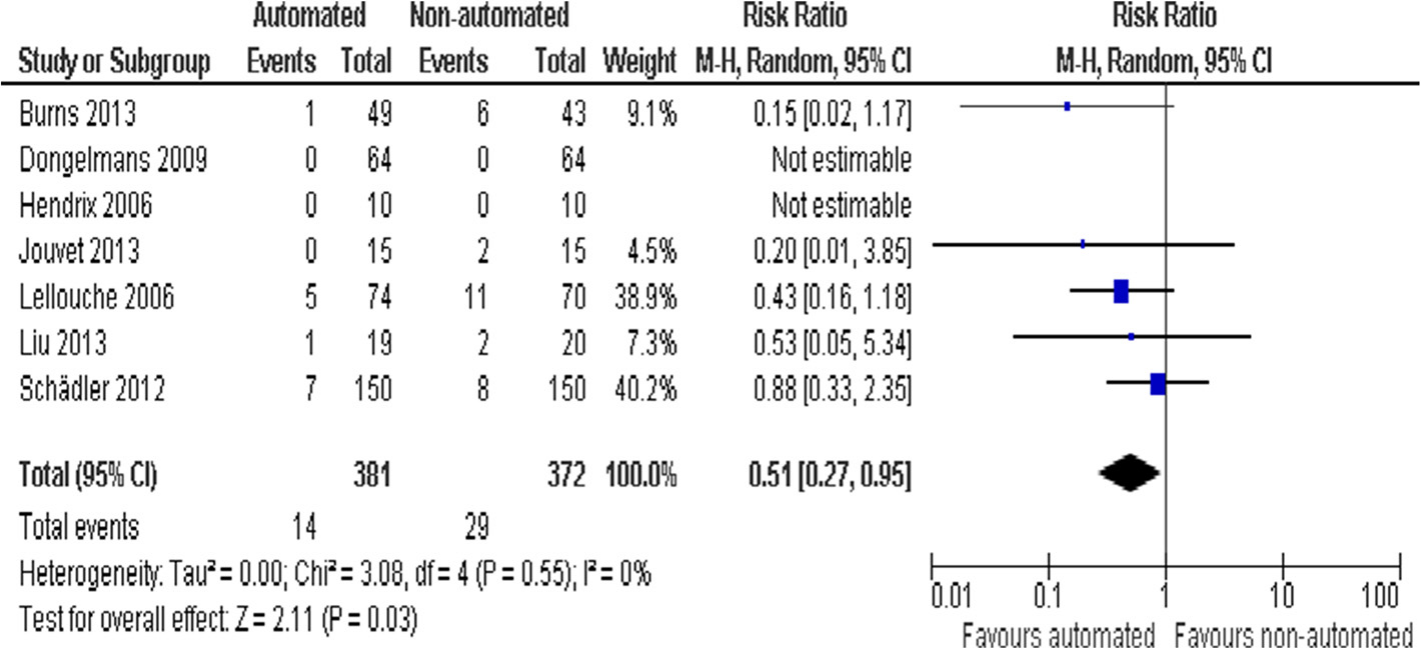
**Prolonged mechanical ventilation.**

**Figure 10:**
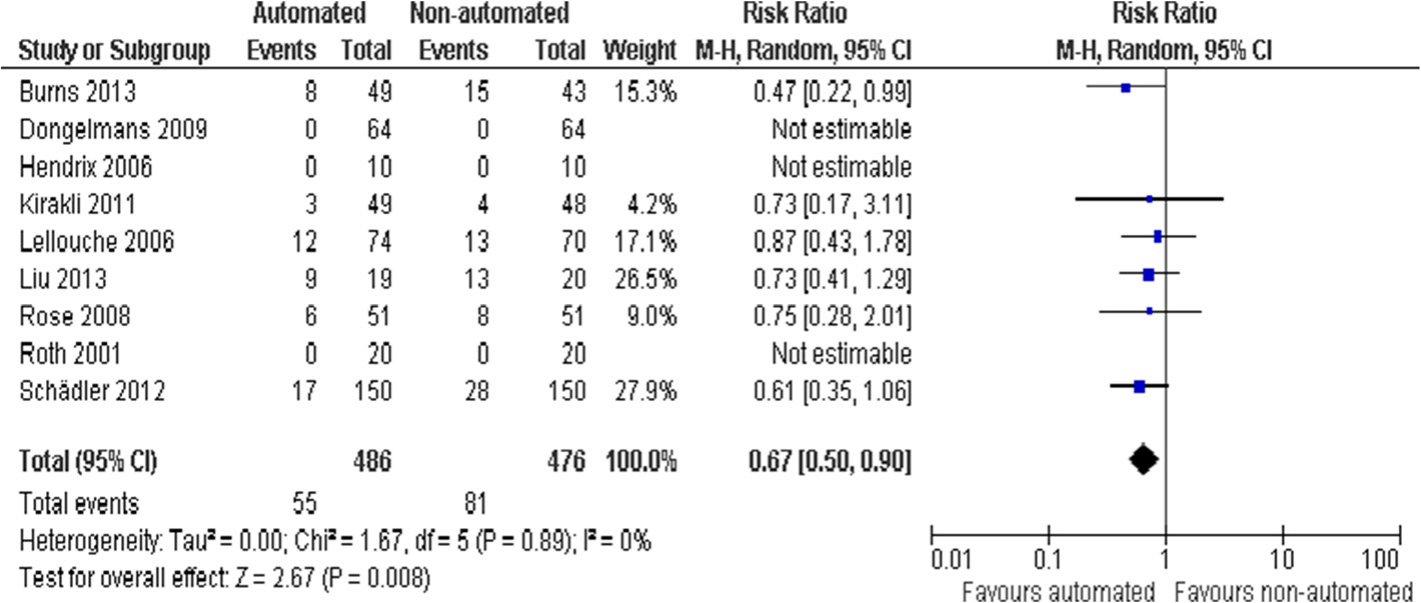
**Tracheostomy.**

We did not find strong evidence that automated systems had an effect on reintubation rates in the 13 trials reporting this outcome (RR 0.80, 0.61 to 1.05, *P* = 0.1), rates of self-extubation (RR 1.24, 0.58 to 2.67, *P* = 0.58; 9 trials), and non-invasive ventilation after extubation (RR 0.73, 0.53 to 1.02, *P* = 0.07; 12 trials). Prolonged mechanical ventilation (RR 0.51, 0.27 to 0.95, *P* = 0.03; 7 trials) (Figure 9), and tracheostomy (RR 0.67, 0.50 to 0.90, *P* = 0.008, 9 trials) were reduced in favour of automated systems (Figure 10).

### Sensitivity analyses

We conducted sensitivity analyses to explore the effects of automated systems prior to log-transforming data. Overall, the pooled weaning duration demonstrated no strong evidence of effect (mean hours −0.75, − 1.85 to 0.34, *P* = 0.18); however, substantial heterogeneity was present (*I*^2^ = 72%, *P* <0.00001). Subanalyses according to ICU population demonstrated reduced weaning duration in mixed/medical ICU populations (mean hours −18.75, −32.30 to −5.20, *P* = 0.007) but not surgical ICU populations (mean hours −0.15, −0.70 to 0.39, *P* = 0.58). Weaning duration was reduced in trials of Smartcare/PS™ (mean hours - 38.46, −58.11 to −18.81, *P* <0.001) but not ASV (mean hours −0.00, −0.07 to 0.06, *P* = 0.98) or other automated systems (mean hours −3.89, −7.71 to 0.07, *P* = 0.05). Weaning duration was reduced in comparison to usual care (mean hours −30.49, −60.63 to −0.35, *P* = 0.05, *I*^2^ = 52%) but not when compared to a protocolized approach. There was no evidence of effect in other measured continuous outcomes. A sensitivity analysis exploring the effect of studies with high risk of bias [27] continued to demonstrate reduced weaning duration using automated systems (mean log hours −0.37, −0.61 to −0.13, *P* = 0.003; 31%, 12% to 46% reduction in geometric mean).

## Discussion

Based on pooled data from 16 eligible trials reporting weaning duration, our study shows automated systems reduced weaning duration by 30% in the geometric mean. Interpretation of the clinical relevance of a 30% reduction in the geometric mean is challenging as the number of hours reduced is dependent on the duration of weaning for a given population. As illustration, if the mean duration of weaning was 24 hours as reported in a large international cohort of ventilated patients [39], a 30% reduction equates to a 7.2 hour reduction in time spent weaning. Subgroup analyses indicated weaning was reduced in trials of mixed/medical ICU populations (42% geometric mean) and Smartcare/PS™ (28% geometric mean) but not in surgical ICU populations despite the inclusion of a 300 participant trial that excluded the typical postsurgical ‘easy-to-wean’ patient. We did not find evidence of effect using automated systems other than Smartcare/PS™. The method of weaning in the trial comparator arms (protocol or non-protocolized usual care) did not influence the effect of automated systems on the duration of weaning. Due to substantial heterogeneity caution must be used when interpreting these results. Automated systems also reduced the time to first extubation, ventilation duration, ICU LOS, tracheostomy and prolonged ventilation. There was no strong evidence of an effect on mortality, reintubation, self-extubation, non-invasive ventilation (NIV) post-extubation or hospital LOS.

When un-logged data were examined, no difference in weaning duration was detected. However, a statistically significant and clinically meaningful (>1 day) difference in weaning duration was found in trials of mixed and medical ICU populations and SmartCare/PS™. Disparate findings in primary and sensitivity analyses are due to trials of surgical patients being given more weight in the unlogged analysis because of differences in relative sizes of the standard deviations meaning the overall conclusion is much closer to zero. It is worth noting that pooled estimates from logged and un-logged data in trials of surgical patients are similar and are the same in mixed and medical ICU populations.

Commercial availability of automated systems has led to growing interest and enhanced feasibility in conducting trials such that 13 of the 21 eligible trials were published in the last five years. Most frequently evaluated systems were Smartcare/PS™ and ASV. While SmartCare/PS™ has an explicit algorithm for pressure support weaning, ASV does not. Another notable difference in these systems is that ASV automates switching from controlled to spontaneous ventilation if the patient’s spontaneous breathing meets the minimum minute ventilation target whereas Smartcare/PS™ requires clinician recognition of spontaneous breathing and activation of Smartcare/PS™. This difference is important when considering the potential impact on weaning and ventilation duration. Systems that can switch automatically from controlled to spontaneous breathing may have more influence on overall ventilation duration than those that rely on clinician activation. These systems may also reduce heterogeneity associated with varying entry criteria for weaning commencement. However, we did not detect an effect on ventilation duration in ASV trials due to inclusion of only surgical ICU patients who generally do not experience protracted weaning and ventilation.

We were unable to conduct subgroup analyses according to the weaning classifications arising from the 2005 consensus conference [2] suggesting these are not yet widely adopted as trial inclusion criteria or *a priori* subgroup analyses. These classifications group patients in terms of number or duration of weaning attempts and may enable better identification of important subgroups or guide patient selection criteria for future trials as opposed to grouping patients according to admission type. We found considerable variation in selection of trial outcomes; indeed, only 71% reported weaning duration. This suggests the need for consensus on a minimum core outcome set for such trials [40]. We identified only two trials in a pediatric population, one in children older than two years due to the age and weight limits imposed by SmartCare/PS™. More than 50% of patients admitted to paediatric ICUs are younger than two years old [41]. This lag in development of automated systems capable of providing appropriate ventilation and weaning to all children probably explains the lack of trials. Given the potential for reduced weaning and ventilation durations, and the small sample size of the two identified trials, there is a need for commercial industry and researchers to further develop and evaluate automated systems adapted to children.

## Conclusions

In conclusion, automated systems may result in clinically meaningful reduced durations of weaning, ventilation and ICU stay. Overall, these systems appear to be safe and can be considered a reasonable approach in the management of ventilator weaning. These potential reductions are more likely to occur in mixed/medical as opposed to surgical ICU populations and with Smartcare/PS™. Due to limited evidence on automated systems other than Smartcare/PS™ and ASV no conclusions can be drawn regarding their influence on outcomes. The method of weaning to which automated systems was compared (protocolized or non-protocolized usual care) did not influence the effect on weaning duration. Due to substantial heterogeneity in trials reporting weaning duration we believe there is a need for an adequately powered, high quality, multi-centre trial in an adult patient population that excludes patients classified as ’simple to wean’ with detailed description of ICU organizational characteristics and weaning/sedation strategies in the comparator arm as these may be contributing to clinical heterogeneity. As little data exists on other automated systems and ASV in prolonged weaning, we are cautious to recommend which should be selected for investigation.

## Key messages

- Automated systems may reduce weaning and ventilation duration particularly for mixed/medical ICU populations.
- These systems may also reduce the need for tracheostomy and prolonged mechanical ventilation.
- Thirteen of the 21 eligible trials were published in the last five years indicating growing interest in automated closed loop systems.
- There is a need for commercial industry and researchers to further develop and evaluate automated systems adapted to children.

## Additional file

Additional file 1:
**Description of automated systems.**

## Abbreviations

ASV: adaptive support ventilation
CI: confidence interval
ICU: intensive care unit
IQR: interquartile range
LOS: length of stay
PAV: proportional assist ventilation
RR: risk ratio
SD: standard deviation
