# Supplementary material for: Automated versus non-automated weaning for reducing the duration of mechanical ventilation for critically ill adults and children: a cochrane systematic review and meta-analysis
Source: Crit Care. 2015 Feb 24;19(1):48. doi: 10.1186/s13054-015-0755-6 (PMC4344786; doi:10.1186/s13054-015-0755-6)
Supplement: Additional file 1: — Description of automated systems. [file 13054_2015_755_MOESM1_ESM.doc]

**Additional File 1: Description of Automated Systems**

1. Smartcare/PS™ (DrägerMedical, Lübeck, Germany) performs closed loop control of pressure support (increases, decreases, or leaves it unchanged) in response to data on the patient’s current respiratory status (respiratory rate, tidal volume (V_T_), and end-tidal carbon dioxide (ETCO_2_)) and its time-course to maintain the patient in a ’respiratory zone of comfort’. The SmartCare/PS system divides weaning into three phases: 1. stabilizing the patient within the respiratory zone of comfort; 2. decreasing pressure support without the patient leaving the comfort zone; 3. testing for extubation readiness by monitoring the patient at the lowest level of pressure support. As opposed to other systems that make breath by breath changes, Smartcare/PS™ changes settings every couple of minutes.

2. Adaptive Support Ventilation (ASV) (HamiltonMedical, Bonaduz, Switzerland) is a closed loop controlled mode of ventilation that adjusts inspiratory pressure and mandatory breath rate on a breath by breath basis to maintain pre-set minimum minute ventilation with an optimal respiratory pattern. Ideal body weight, percentage of minute ventilation desired, and maximal inspiratory pressure are selected on initiation of ASV by the clinician. ASV is delivered as pressure- controlled ventilation (PCV) and calculates optimal tidal volume and respiratory rate targets using the Otis formula and based on the pre-set minimum minute ventilation, theoretical dead space calculated from the ideal body weight, and the expiratory time constant. When the patient makes an inspiratory effort, ASV switches from PCV to pressure support ventilation (PSV). Pressure support is continuously adapted to the patient’s respiratory rate and V_T_ to achieve the desired minute ventilation.

3. Automode (Siemens, Solna, Sweden) uses an algorithm to switch from a controlled mode, for example PCV, to a support mode such as PSV based on detection of patient triggering of two consecutive breaths. The mode is switched from support to control when the patient experiences prolonged apnoea (> 12 seconds). Other possible mode switches are from volume controlled ventilation to volume support ventilation (VSV) or from pressure regulated volume control to VSV.

4. Proportional Assist Ventilation (PAV+) automatically adjusts airway pressure based on measurement of compliance and resistance throughout the inspiratory cycle to maintain an appropriate degree of support. There are no set targets for pressure, volume, or flow, rather airway pressure is increased or decreased in proportion to patient effort via a positive feedback control using respiratory elastance and resistance as feedback signals. The patient’s respiratory drive determines the respiratory rate and inspiratory time.

5. Mandatory Minute Ventilation (MMV) (Dräger Medical, Lübeck, Germany) uses closed loop control of the mandatory breath rate while considering the patient’s spontaneous breath rate based on a clinician predetermined minute ventilation. All other ventilator parameters are clinician selected. The mandatory breath rate is variable, dependent on the patient’s respiratory drive. Patients able to breath spontaneously above the predetermined minute ventilation essentially receive PSV; patients experiencing apnea receive controlled ventilation.

6. Proportional Pressure Support (PPS) (DrägerMedical, Lübeck, Germany) is based on the same principals as PAV. In conventional PSV, pressure support is delivered as a fixed pressure during each inspiratory phase. In PPS, pressure support is provided proportionately to the work of breathing that alters due to changes in airway resistance and lung compliance.

7. Neurally Adjusted Ventilatory Assist (NAVA) (Maquet, Solna, Sweden) delivers partial ventilatory support via a feedback loop generated through monitoring of neural inspiratory activity using continuous oesophageal recording of the diaphragmatic electromyogram.

Ventilatory support is delivered in proportion to the signal’s intensity and cycled on and off according to its time course.

8. Intellivent-ASV^Ⓡ^(Hamilton Medical, Rhäzüns, Switzerland) is a relatively new extension of ASV that uses closed loop control to adjust minute ventilation based on the ETCO_2_ and oxygenation by automatically adjusting the fraction of inspired oxygen (FiO_2_) and positive end-expiratory pressure (PEEP) in combination based on the Acute Respiratory Distress SyndromeNetwork (ARDSnet) PEEP-FiO_2_ table.

9. Mandatory Rate Ventilation (MRV) (Taema-Horus Ventilator ® Air Liquide, France) uses closed loop control to adjust pressure support based on a respiratory rate target. The ventilator compares the average respiratory rate over four respiratory cycles to the target. If the average respiratory rate is higher than the target, pressure support is increased by 1 cm H_2_O, if lower the pressure support is decreased by 1 cm H_2_O.

References

1. Dojat M, Brochard L, Lemaire F, Harf A: A knowledge-based system for assisted ventilation of patients in intensive care units. *Int J Clin Monit Comput* 1992, 9:239-250.

2. Sulzer CF, Chiolero R, Chassot PG, Mueller XM, Revelly JP: Adaptive support ventilation for fast tracheal extubation after cardiac surgery: a randomized controlled study. *Anesthesiol* 2001, 95:1339-1345.

3. Otis A, Fenn W, Rahn H: Mechanics of breathing in man. *Journal of Applied Physiology* 1950, 2:592-607.

4. Roth H, luecke T, Lansche G, Bender H, Quintel M: Effects of patient-triggered automatic switching between mandatory and supported ventilation in the postoperative weaning patients. *Intensive Care Medicine* 2001, 27(1):47-51.

5. Kondili E, Prinianakis G, Alexopoulou C, Vakouti E, Klimathianaki M, Georgopoulos D: Respiratory load compensation during mechanical ventilation-proportional assist ventilation with load-adjustable gain factors versus pressure support. *Intensive Care Med* 2006, 32:692-699.

6. Branson R, Johannigman J: What it the evidence base for the newer ventilation modes? *Respiratory Care* 2004, 49(7):742-760.

7. Hewlett AM, Platt AS, Terry VG: Mandatory minute volume. A new concept in weaning from mechanical ventilation. *Anaesthesia* 1977, 32:163-169.

8. Younes M: Proportional assist ventilation, a new approach to ventilatory support: theory. *Am Rev Respir Dis* 1992, 145:114-120.

9. Sinderby C, Navalesi P, Beck J, Skrobik Y, Comtois N, Friberg S, Gottfried S, Lindstrom L: Neural control of mechanical ventilation in respiratory failure. *Nat Med* 1999, 5:1433–1436.

10. Schmidt M, Demoule A, Cracco C, Gharbi A, Fiamma M-N, Straus C, Duguet A, Gottfried SB, Similowski T: Neurally adjusted ventilatory assist increases respiratory variability and complexity in acute respiratory failure. *Anesthesiology* 2010, 112(3):670-681.

11. Arnal J-M, Wysocki M, Novotni D, Demory D, Lopez R, Donati S, Granier I, Corno G, Durand-Gasselin J: Safety and efficacy of a fully closed loop control ventilation (Intellivent-ASVⓇ) in sedated ICU patients with acute respiratory failure: a prospective randomized crossover study. *Intensive Care Med* 2012, 38:781-787.

12. Taniguchi C, Eid R, Saghabi C, Souza R, Silva E, Knobel E, Paes A, Barbas C: Automatic versus manual pressure support reduction in the weaning of post-operative patients: a randomised controlled trial. *Crit Care* 2009, 13:R6.
